# Clinical effect and prognostic factor of electric stimulation and biofeedback therapy on postpartum pelvic organ prolapse

**DOI:** 10.1590/1980-220X-REEUSP-2023-0421en

**Published:** 2024-07-19

**Authors:** Hongjin Wu, Xiaoying Zhong, Linqian He, Xixi Li, Yan Zeng, Yuanli Jia

**Affiliations:** 1 Chengdu Medical College, School of Nursing, Chengdu, Sichuan, China.; 2 University of Electronic Science and Technology of China, School of Medicine, Mianyang Central Hospital, Obstetrics and Gynecology Department, Mianyang, Sichuan, China.; 3 University of Electronic Science and Technology of China, School of Medicine, Department of Nursing, Mianyang Central Hospital, Mianyang, Sichuan, China.

**Keywords:** Pelvic Organ Prolapse, Postpartum Period, Electric Stimulation, Biofeedback, Psychology, Prolapso de Órgão Pélvico, Período Pós-Parto, Estimulação Elétrica, Biorretroalimentação Psicológica, Prolapso de Órgano Pélvico, Periodo Posparto, Estimulación Eléctrica, Biorretroalimentación Psicológica

## Abstract

**Objective:**

To explore the effects of electric stimulation and biofeedback therapy in patients with postpartum pelvic organ prolapse and to identify factors that can affect therapeutic efficacy outcomes.

**Method:**

This retrospective study analysed clinical data about patients with postpartum pelvic organ prolapse. A total of 328 women with pelvic organ prolapse at 6 weeks postpartum were recruited from one tertiary hospitals in Sichuan province in China, between March 2019 and March 2022. The prognostic factors of therapeutic efficacy were analysed using logistic regression and decision tree model.

**Results:**

Overall, 259 women showed clinical benefits from the treatment. The logistic regression model showed that parity, pelvic floor muscle training at home, and the pelvic organ prolapse quantitation stage before treatment were independent prognostic factors. The decision tree model showed that the pelvic organ prolapse quantitation stage before treatment was the main prognostic factor, followed by parity. There was no significant difference in the area under the receiver operating characteristic curve between the two models.

**Conclusion:**

Parity, pelvic floor muscle training at home, and the pelvic organ prolapse quantitation stage before treatment were important prognostic factors of electric stimulation and biofeedback therapy on postpartum pelvic organ prolapse.

## RESUMO

**Objetivo:**

Explorar o efeito da estimulação elétrica e da terapia de *biofeedback* em pacientes com prolapso de órgãos pélvicos pós-parto e identificar fatores que podem afetar os resultados da eficácia terapêutica.

**Método:**

Este estudo é uma análise retrospectiva de dados clínicos de pacientes com prolapso de órgãos pélvicos pós-parto. Um total de 328 mulheres com prolapso de órgãos pélvicos nas seis semanas pós-parto foram recrutadas em um hospital terciário na província de Sichuan, na China, entre março de 2019 e março de 2022. Os fatores prognósticos de eficácia terapêutica foram analisados por meio de regressão logística e modelo de árvore de decisão.

**Resultados:**

No geral, 259 mulheres apresentaram benefícios clínicos com o tratamento. O modelo de regressão logística mostrou que a paridade, o treinamento muscular do assoalho pélvico em casa e o estágio de quantificação do prolapso de órgãos pélvicos antes do tratamento foram fatores prognósticos independentes. O modelo de árvore de decisão mostrou que o estágio de quantificação do prolapso de órgãos pélvicos antes do tratamento foi o principal fator prognóstico, seguido pela paridade. Não houve diferença significativa na área sob a curva ROC entre os dois modelos.

**Conclusão:**

A paridade, o treinamento muscular do assoalho pélvico em casa e o estágio de quantificação do prolapso de órgãos pélvicos antes do tratamento foram importantes fatores prognósticos da estimulação elétrica e da terapia de *biofeedback* no prolapso de órgãos pélvicos pós-parto.

## RESUMEN

**Objetivo:**

Explorar el efecto de la estimulación eléctrica y la terapia de biorretroalimentación en pacientes con prolapso posparto de órganos pélvicos e identificar los factores que pueden afectar los resultados de la eficacia terapéutica.

**Método:**

Este estudio es un análisis retrospectivo de los datos clínicos de pacientes con prolapso posparto de órganos pélvicos. Entre marzo de 2019 y marzo de 2022, se reclutaron un total de 328 mujeres con prolapso de órganos pélvicos en las seis semanas posteriores al parto en un hospital terciario de la provincia de Sichuan, China. Los factores pronósticos de eficacia terapéutica se analizaron mediante regresión logística y el modelo de árbol de decisión.

**Resultados:**

En total, 259 mujeres mostraron beneficios clínicos relacionados con el tratamiento. El modelo de regresión logística mostró que la paridad, el entrenamiento en casa de la musculatura del suelo pélvico y la etapa de cuantificación del prolapso de órganos pélvicos antes del tratamiento fueron factores pronósticos independientes. El modelo de árbol de decisión mostró que la etapa de cuantificación del prolapso de órganos pélvicos previa al tratamiento fue el principal factor pronóstico, seguido de la paridad. No hubo diferencias significativas en el área bajo la curva ROC entre los dos modelos.

**Conclusión:**

La paridad, el entrenamiento en casa de la musculatura del suelo pélvico y la etapa de cuantificación del prolapso de órganos pélvicos previa al tratamiento fueron factores pronósticos importantes de la estimulación eléctrica y la terapia de biorretroalimentación en el prolapso posparto de órganos pélvicos.

## INTRODUCTION

Postpartum pelvic organ prolapse (POP) is a common clinical condition where pelvic organs move down due to a weakness or defect in the pelvic floor support structures after childbirth. Patients with postpartum POP often have different degrees of urinary, defaecatory, and sexual dysfunction, which seriously affect women’s quality of life, mental health, dignity, and sexual satisfaction^(1,2,3)^. It is estimated that the prevalence of POP will increase by approximately 50% in the United States by 2050^(4)^. The incidence rate among women aged >50 years is as high as 40%^(5,6)^. Early pelvic floor rehabilitation intervention was a crucial step to improve women’s quality of life during the postpartum period.

Currently, the treatment protocols for POP include surgery and conservative treatment^(7)^. Surgical treatment may increase the risk of postoperative complications and prolapse recurrence. POP Quantification (POP-Q) is a standardized system used to measure and describe the severity of pelvic organ prolapse^(8)^. It provides a consistent way for healthcare professionals to assess and communicate the extent of prolapse in women. The severity of pelvic organ prolapse can be classified into stage 0 (no prolapse), stage I (mild), stage II (moderate), stage III (severe), and stage IV (prolapse outside the vagina). If the degree of prolapse is slight or there is no surgical indication, conservative treatment is usually recommended for rehabilitation^(9)^. At present, women with mild postpartum POP mainly receive non-surgical treatment, including electric stimulation and biofeedback therapy^(10,11)^.

Previous studies have suggested that electric stimulation combined with biofeedback therapy can improve pelvic floor muscle contraction, POP symptoms, level of sexual function, and vaginal relaxation, and can ease sexual difficulties^(12,13,14)^. In China, however, there is lack of information available on the clinical effects of electric stimulation combined with biofeedback therapy on postpartum POP. In this study, we collected information on patients with postpartum POP through established retrospective data on postpartum women to determine the clinical effects and prognostic factors of electric stimulation combined with biofeedback therapy in Chinese patients with postpartum POP.

## METHODS

### Design and Setting

We retrospectively analysed the data of patients who were diagnosed with postpartum POP from one tertiary hospital in Sichuan province in China, between March 2019 and March 2022.

The pelvic floor rehabilitation instrument (PHENIX USB 4; Vivaltis, Montpellier, France) was used to treat the patients with electric stimulation and biofeedback therapy. Electric stimulation and biofeedback treatments was administered for 30 minutes twice a week for 15 sessions, all operated by two pelvic floor specialist nurses. Before the first treatment, a nurse with professional training conducted an electrophysiological examination for each patient. Prior to the treatment, patients voided themselves of stool and urine. Subsequently, patients were asked to adopt a half-lying position and separate their legs. After lubricating the vaginal electrode with sterile lubricant, it was placed in the vagina, and the current intensity was gradually increased from 0 mA until the patient felt the current stimulation. It is appropriate for the patient to feel that the pelvic floor muscles have obvious contraction, but without any obvious discomfort. Electric stimulation lasted for 20 minutes each time. Afterward, the patients relaxed naturally and performed vaginal muscle contractions without abdominal pressure. According to the graphic display of the therapeutic instrument, two modes of vaginal muscle training were performed alternately: one in which the vaginal muscles were contracted and immediately relaxed, and the other in which the vaginal muscles were contracted for a period of 5 seconds and then relaxed for a total of 10 minutes.

At the end of the first pelvic floor rehabilitation treatment, a pelvic floor specialist nurse instructed the patient one-on-one on the correct pelvic floor muscle training (PFMT) exercises and forwards the pelvic floor exercise video to the patient through the public website. The specific method is to empty the bladder and relax before the exercise, and step on the floor with legs spread. Subsequently, the patient should adjust the anal contraction according to the rhythm of breathing, contracting when inhaling and relaxing when exhaling. Each contraction lasts no less than 5 seconds, followed by relaxation, and is repeated for 10–15 minutes, 3 times a day.

### Population, Inclusion and Exclusion Criteria

Patients were eligible for inclusion based on these criteria^(15,16,17,18)^: 1) complete clinical data available; and 2) full-term singleton pregnancy. We excluded patients who had: 1) a history of pelvic injury and pelvic surgery; and 2) organic diseases of the reproductive tract. Demographic data were extracted from medical record system, including age, education, parity, number of pregnancies, number of abortions, type of delivery, neonatal weight classification, pre-pregnancy body mass index (BMI), PFMT done at home, clinical effects, and the POP-Q stages before and after treatment. A total of 343 women with POP at 6 weeks postpartum were diagnosed. We excluded 15 women owing to missing clinical data; therefore, a total of 328 women with POP at 6 weeks postpartum were included for data analysis.

### Data Grouping

The electric stimulation and biofeedback therapy efficacy evaluation criteria were as follows^(19)^:

(1) Cure, namely, the POP stage changing to stage 0 from stage I and stage II after treatment.
(2) Remarkable efficiency, namely, the POP stage changing to stage I from stage II after treatment.
(3) Invalid, namely, the POP stage not changing after treatment.
(4) Cure and remarkable efficiency were regarded as effective.

### Statistical Analysis

Data were recorded in Excel 2013 (Microsoft Corp., Redmond, WA, USA) version for data entry and sorting. The data were checked by two people and analysed by SPSS version 29.0 (IBM Corp., Armonk, NY, USA). Frequency, component ratio, mean and standard deviation were used to make descriptive statistics of demographic data. The Logistic regression analysis model and the decision tree model were established, and the influencing factors of the two models were compared^(20)^. In the decision tree model, classification and regression tree algorithm was used for analysis, all independent variables were included into the decision tree model, the minimum sample size in the parent and child nodes is 100 and 50, respectively, and the maximum tree depth is 3^(21,22)^. The test level of separation and merger α was 0.05. The Logistic regression model used the presence or absence of cognitive impairment as the dependent variable, and included the independent variables with statistical significance in the univariate analysis to establish the Logistic regression model. P < 0.05 was considered statistically significant. Hosmer–Lemeshow goodness of fit test, overall prediction accuracy, model risk statistics and receiver operating characteristic were used for the overall results; the two models’ curve, specificity, and sensitivity were evaluated.

### Ethical Approval

All methods were performed in accordance with the relevant guidelines and regulations or in accordance with the Declaration of Helsinki. The Ethics Committee approval of the Mianyang Central Hospital, School of Medicine, University of Electronic Science and Technology of China approved the study protocol before conducting the study (ID: S20230306-02). This study was approved by the Ethics and Clinical Investigation Committee of Hospital, with exemption granted on the need for informed consent.

## RESULTS

### Demographic Characteristics of Participants

A total of 328 women with POP at 6 weeks postpartum were analysed. Most women were aged <35 years (81.7%), had university education (72%), and were primipara (67.7%). A summary of the demographic data is shown in Table 1.

**Table 1:** Demographic characteristics of participants (n = 328) – Mianyang, Sichuan, China, 2023.

Variables	Number (n)	Percentage (%)
**Age (years)**		
<35	268	81.7
≥35	60	18.3
**Education**		
Junior high school and below	20	6.1
Senior high school	49	14.9
University	236	72.0
Master and above	23	7.0
**Parity**		
Primipara	222	67.7
Multipara	106	32.3
**Number of pregnancies**		
1	143	43.6
2	91	27.7
≥3	94	28.7
**Number of abortions**		
None	199	60.7
1	72	22
≥2	57	17.4
**Type of delivery**		
Vaginal delivery	189	57.6
Caesarean delivery	139	42.4
**Neonatal weight classification**		
Low weight infant	11	3.4
Normal weight infant	299	91.2
macrosomia	18	5.5
**Pre-pregnancy BMI**		
Thinnish	125	38.1
Normal	196	59.8
Overweight	5	1.5
Obesity	2	0.6
**PFMT done at home**		
Yes	296	90.2
None	32	9.8
**Clinical effect**		
effective	259	79.0
invalid	69	21.0
**POP-Q stage (before treatment)**		
Stage 0	–	–
Stage I	194	59.1
Stage II	134	40.9
**POP-Q stage (after treatment)**		
Stage 0	154	47
Stage I	154	47
Stage II	20	6.0

Note: SD, standard deviation; BMI, body mass index; PFMT, pelvic floor muscle training; POP-Q, Pelvic Organ Prolapse Quantification.

### Univariate Analysis Prognostic Factor of Electric Stimulation Combined with Biofeedback Therapy on Postpartum Pop Patients

There were significant differences in parity, PFMT at home, and the POP-Q stage before treatment between the effective treatment group and the invalid treatment group (P < 0.05). The univariate analysis of prognostic factors for electric stimulation combined with biofeedback therapy on postpartum POP is shown in Table 2.

**Table 2:** Univariate analysis of prognostic factors for electric ­stimulation and biofeedback therapy on patients with postpartum POP (n = 328) – Mianyang, Sichuan, China, 2023.

Variables	Clinical effect		χ^2^	*P*
	Effective (n = 259)	Invalid (n = 69)		
**Age (years)**				
<35	208	60	1.611	0.204
≥35	51	9		
**Education**				
Junior high school and below	14	6	2.637	0.451
Senior high school	42	7		
University	184	52		
Master and above	19	4		
**Parity**				
Primipara	167	55	5.779	0.016*
Multipara	92	14		
**Number of pregnancies**				
1	106	37	3.581	0.167
2	75	16		
≥3	78	16		
**Number of abortions**				
None	153	46	1.572	0.456
1	58	14		
≥2	48	9		
**Type of delivery**				
Vaginal delivery	146	43	0.789	0.374
Caesarean delivery	113	26		
**Neonatal weight classification**				
Low weight infant	9	2	0.69	0.996
Normal weight infant	236	63		
macrosomia	14	4		
**Pre-pregnancy BMI**				
Thinnish	99	26	0.558	0.906
Normal	154	42		
Overweight	4	1		
Obesity	2	—		
**PFMT done at home**				
Yes	248	48	42.44	<0.001**
None	11	21		
**POP-Q stage (before treatment)**				
Stage 0	—	—	19.907	<0.001**
Stage I	137	57		
Stage II	122	12		

Note: SD, standard deviation; BMI, body mass index; PFMT, pelvic floor muscle training; POP, Pelvic Organ Prolapse; POP-Q, Pelvic Organ Prolapse Quantification; *P < 0.05; **P < 0.001.

### Logistic Regression Analysis of Prognostic Factor for Electric Stimulation Combined with Biofeedback Therapy on Patients with Postpartum Pop

With the clinical effect as the dependent variable and the variables with statistical significance in the univariate analysis as independent variables, logistic regression analysis was performed. The results showed that parity, PFMT at home, and the POP-Q stage before treatment were independent risk factors for electric stimulation combined with biofeedback therapy on postpartum POP (see Table 3).

**Table 3:** Logistic regression analysis of prognostic factor for electric stimulation and biofeedback therapy on patients with postpartum POP (n = 328) – Mianyang, Sichuan, China, 2023.

Variables	*B*	*SE*	*Wald χ* ^2^	*P*	*OR*	95% *CI*
Constant	0.644	0.484	1.768	0.184	1.904	
Parity	–0.816	0.367	4.934	0.026	0.442	0.215–0.909
PFMT done at home	2.58	0.455	32.122	<0.001	13.2	5.408–32.219
POP-Q stage before treatment	–1.616	0.384	17.748	<0.001	0.199	0.094–0.421

Note: *B*, Unstandardized Coefficient; SE, Standard Error; Hosmer–Lemeshow test *χ* ^2^ = 3.816, P = 0.282; Cox and Snell R^2^ = 0.175; Nagelker ‘ke R^2^ = 0.272; Parity (Primipara = 0, Multipara = 1); PFMT done at home (Yes = 0, None = 1); POP-Q stage before treatment (Stage 0 = 0, Stage I = 1, Stage II = 2); POP, pelvic organ prolapse; *SE*, standard error; *OR*, odds ratio; *CI*, confidence interval.

### Decision Tree Model Analysis of Prognostic Factors for Electric Stimulation Combined with Biofeedback Therapy on Patients with Postpartum Pop

**Figure 1:**
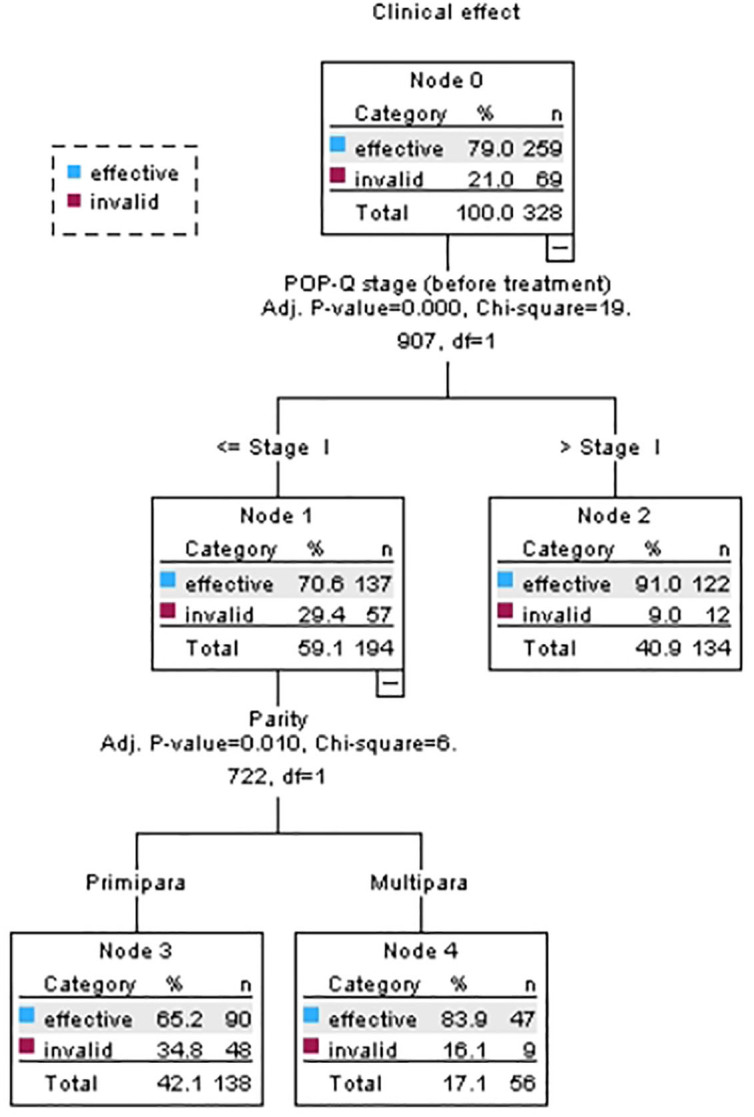
Decision tree model of prognostic factor of electric stimulation and biofeedback therapy on patients with postpartum POP. Mianyang, Sichuan, China, 2023.

The prediction tree diagram is shown in Figure 1. The tree consists of two layers with a total of six nodes and three terminal nodes. The POP-Q stage before treatment and parity were the variables affecting the clinical effect of electric stimulation combined with biofeedback therapy on postpartum POP. A total of three classification rules were extracted: (1) women with POP-Q stage (before treatment) >I accounted for 91.00% of the node composition; (2) women with POP-Q stage (before treatment) ≤I and who were primipara accounted for 65.20% of the node composition; and (3) women with POP-Q stage (before treatment) ≤I and who were multipara accounted for 83.90% of the node composition.

### Comparison of the Results of the Two Models

The influencing factors were ranked according to the absolute value of β in the logistic regression model and the position and χ^2^ value of the influencing factors in the decision tree model. In the logistic regression model, the three factors that had a greater impact on the clinical effects of electric stimulation combined with biofeedback therapy on postpartum POP were: parity (β = –0.816), PFMT at home (β = 2.58), and the POP-Q stage before treatment (β = –1.616). The two factors in the decision tree model were parity and POP-Q stage before treatment. There were differences in the analysis results of the two models on prognostic factor of electric stimulation combined with biofeedback therapy on postpartum POP.

The area under the receiver operating characteristic (ROC) curve of the logistic regression model was 0.779 (95% confidence interval [CI]: 0.717–0.840), with a sensitivity of 0.957 and a specificity of 0.834. The area under the ROC curve of the decision tree model was 0.689 (95% CI: 0.621–0.757) with a sensitivity of 0.826 and a specificity of 0.529 (Table 4).

**Table 4:** Comparison of prediction effect between logistic regression and decision tree models – Mianyang, Sichuan, China, 2023.

Model	*AUC*	*SE*	*P*	95% *CI*
Logistic regression	0.779	0.031	<0.001	0.717–0.840
Decision tree model	0.689	0.035	<0.001	0.621–0.757

Note: *AUC*, area under the curve; *SE*, standard Error; *CI*, confidence interval.

## DISCUSSION

Electric stimulation and biofeedback therapy is the most common pelvic floor rehabilitation therapy in clinical practice. Electric stimulation therapy provides pulse currents to the vagina to regulate the excitability of innervated muscle, making muscle tissue contract and relax passively, thus improving pelvic floor muscle strength^(11)^. Biofeedback therapy measures vaginal contractility through the pressure sensor in the vagina, provides visual feedback signals, intuitively informs patients of the active contractility of pelvic floor muscles, promotes patients to correctly perform muscle contractions, and effectively promotes the recovery of patients’ neurological function^(13)^. The findings of this study demonstrated that the effect of electric stimulation combined with biofeedback therapy is significant for postpartum POP.

In line with previous literature, it was found that the clinical effects of electric stimulation combined with biofeedback therapy on postpartum POP was linked to parity; primipara women reported a 44.2% increase in effectiveness compared with multipara ones. The physical changes that occur during pregnancy and the stress placed on the pelvic floor during childbirth can weaken the muscles and tissues in the area, leading to dysfunction. Previous studies have found that pregnancy and childbirth are high-risk factors for postpartum POP.

This study found that women who adhered to rehabilitation at home had a 20.0% increase in effectiveness compared with those who did not, consistent with the results of Resende et al.^(23)^. Studies have shown that persistence in PFMT for women with mild POP can improve the prolapse and its symptoms^(24,25,26)^. PFMT can contract pelvic floor muscles and make their structure conducive to maintaining the normal anatomical positions of pelvic organs. Pelvic floor exercise can strengthen the pelvic floor muscles to support and combat an increase of abdominal pressure^(27)^. Notably, we observed statistically significant differences between clinical effects and POP-Q, where women with lower POP-Q stages had a 19.9% increase in effectiveness compared to those with higher POP-Q stages. Previous studies have shown that the severity of prolapse, as determined by POP-Q, can influence the clinical effect of pelvic floor rehabilitation.

There were some limitations to this study. First, retrospective studies rely on existing medical records, which may be incomplete or inaccurate, leading to biased results. Second, in this study, data on patients with postpartum POP were available from only one hospital, and there may have been some selection bias in the study population, resulting in limited representativeness. Third, retrospective studies can only establish associations between variables, not causality. Further research, such as prospective studies or randomized controlled trials, is needed to establish the prognostic factors for electric stimulation combined with biofeedback therapy for postpartum POP.

## CONCLUSION

This study showed that parity, PFMT at home, and the POP-Q stage before treatment were important prognostic factors for electric stimulation combined with biofeedback therapy in patients with postpartum POP.
